# Supplementary material for: Breeding of High Cooking and Eating Quality in Rice by Marker-Assisted Backcrossing (MABc) Using KASP Markers
Source: Plants (Basel). 2021 Apr 19;10(4):804. doi: 10.3390/plants10040804 (PMC8073074; doi:10.3390/plants10040804)
Supplement: Supplementary file 1 [file plants-10-00804-s001.zip › plants-1181105-SI.pdf]

plants MDPI

Table S1. Information about markers used in foreground selection.

Marker	Ori.	Seq.	Product Size (bp)	Tm (°C)
SSIIIa_1st	Fw<br>Rv	CTAGGGGATATGCTCCGAGA<br>TCCACCTCGAGTGTGTCTTC	661	58
SSIIIa_2nd	Fw	ACACTCTTTCCCTACACGACGCTCTTCCGATCT**TT-GCCCAGAAATTTGTTGAA**	202	59
	Rv	GTGACTGGAGTTCAGACGTGTGCTCTTCCGATCT**CCACTGCAGCAC-TATGCAAT**		

Table S2. Genes identified with SNP and amino acid variation in mutant region between Samgwang and Milkyqueen.

No.	Gene ID	SNP Position(bp)	Reference	Allele	Amino Acid Mutation	Gene Description
1	Os08g0143400	2,388,554	T	C	I(T) / T(C)	SWIRM and amine oxidase domain-containing protein
2	Os08g0146001	2,584,408	G	T	T(G,T)	Hypothetical conserved gene
3	Os08g0149000	2,808,619	G	T	E(G) / D(T)	Conserved hypothetical protein
		2,810,430	T	C		
		2,810,464	G	A		
		2,810,468	G	T		
		2,810,575	T	C		
		2,810,613	G	A		
		2,810,859	C	T		
		2,811,267	C	T		
		2,811,434	A	G		
		2,811,593	A	T		
		2,811,663	T	G		
		2,811,874	A	T		
		2,811,938	T	G		
4	Os08g0149100	2,817,794	C	A	P(C,A)	Conserved hypothetical protein
5	Os08g0151300	2,948,776	C	T	P(C) / L(T)	R2R3-MYB transcription factor
		2,949,645	G	A		
6	Os08g0152333	3,000,973	T	C		Hypothetical protein
		3,001,197	T	C	R(T,C)	
		3,001,357	A	T	I(A,T)	
7	Os08g0190500	5,315,164	C	A	L(C) / I(A)	Conserved hypothetical protein
8	Os08g0191433	5,354,128	G	A	A(G) / T(A)	Starch synthase
		5,354,184	C	T	I(C,T)	
		5,354,380	T	C	I(T) / T(C)	
		5,354,592	C	T	I(C,T)	
		5,354,722	C	T	I(C,T)	
		5,354,862	G	A	Q(G,A)	
		5,355,177	G	A	A(G) / T(A)	
		5,355,240	G	A	Q(G,A)	
		5,355,283	C	T	P(C) / S(T)	
		5,355,378	G	A	Q(G,A)	
		5,355,495	T	A	I(T) / N(A)	
		5,355,883	A	T	L(A,T)	
		5,356,488	G	A	L(G,A)	
		5,356,597	A	T		
		5,356,600	A	G		
		5,356,846	C	T	R(C) / W(T)	
		5,356,961	G	A	E(G,A)	

		5,357,030	T	A		
		5,357,125	T	C		
		5,357,151	A	G		
		5,357,214	A	C		
		5,357,619	G	A	A(G) / T(A)	
		5,358,029	A	G		
		5,358,190	A	G		
		5,358,308	C	T		
		5,358,346	T	C	I(T,C)	
		5,358,518	A	T		
		5,358,803	C	T		
8	Os08g0191433	5,358,899	G	A	I(T,C)	Starch synthase
		5,358,962	C	T		
		5,358,990	A	G		
		5,358,999	C	T		
		5,359,102	C	T		
		5,359,108	T	C		
		5,359,983	A	G		
		5,360,061	C	A		
		5,360,200	T	G		
		5,360,263	T	G		
		5,360,399	A	C		
		5,360,595	T	A		
		5,360,623	G	A		
		5,360,992	G	A		
9	Os08g0191466	5,361,571	T	C		Hypothetical gene
		5,361,621	T	C		
10	Os08g0191700	5,374,282	C	T		Glyoxalase I
		5,374,524	C	T		
		5,375,967	T	C		
		5,376,876	G	C		
		5,376,958	C	G	A(C) / G(G)	
19	Os09g0270700	5,278,515	T	C	T(T,C)	Disease resistance protein domain containing protein
20	Os09g0271000	5,322,883	T	A	M(T) / K(A)	Hypothetical protein
21	Os09g0272900	5,473,217	A	G	-	Disease resistance protein domain containing protein
		5,479,708	G	C	K(G) / N(C)	
		5,480,407	A	G	N(A) / S(G)	
22	Os09g0273600	5,538,837	T	C	A(G) / P(C) / S(T)	Hypothetical gene
23	Os09g0277100	5,758,819	G	A	P(C) / T(A) / A(G)	Hypothetical protein
24	Os09g0278300	5,799,059	T	C	A(C) / V(T)	Serine hydroxymethyltransferase domain containing protein
		5,805,200	G	A	-	
25	Os09g0279300	5,865,094	C	T	I(C,T)	Mitochondrial inner membrane translocase
26	Os09g0279400	5,866,891	A	G	R(A) / G(G)	Rhodanese-like domain containing protein
		5,868,122	C	T	-	
		5,868,279	G	A	S(G) / N(A)	
		5,868,331	G	A	E(G,A)	
27	Os09g0292300	6,803,268	T	A	L(T) / H(A)	Myb/SANT-like domain domain containing protein
		6,805,086	C	T	C(C,T)	

28	Os09g0292900	6,873,519	G	T	A(G,T)	Galactose oxidase
		6,873,637	C	A	L(C) / I(A)	
		6,873,835	A	G	T(A) / A(G)	
29	Os09g0295850	7,162,946	A	G	I(A) / V(G)	Conserved hypothetical protein
		7,162,960	C	T	C(C,T)	
		7,163,042	T	C	V(T) / H(C)	
30	Os09g0296450	7,167,405	G	T	-	Hypothetical protein
		7,167,618	G	A	-	
		7,168,380	A	C	-	
		7,169,920	C	T	E(A) / D(T,C)	
31	Os09g0296700	7,180,181	G	T	R(G) / L(T)	Glycosyl transferase
		7,181,431	C	T	R(C) / W(T)	
32	Os09g0296900	7,191,217	A	G	T(A) / A(G)	WD40 repeat-like domain containing protein
		7,191,938	T	C	W(T) / R(C)	
		7,192,549	A	T	-	
		7,193,810	A	G	T(A) / A(G)	
		7,194,521	T	A	-	
		7,194,743	C	A	-	
33	Os09g0297000	7,199,809	G	T	-	Similar to Ferrochelatase
33	Os09g0297000	7,202,574	A	G	-	Similar to Ferrochelatase
		7,204,241	A	C	A(A,C)	
34	Os09g0297100	7,206,737	G	A	-	Similar to CROC-1-like protein
		7,206,823	T	G	-	
		7,206,941	C	T	R(C) / C(T)	
		7,206,956	G	A	I(A) / V(G)	
		7,207,398	C	A	-	
		7,207,651	A	C	-	
		7,207,745	C	G	Q(C) / E(G)	
35	Os09g0297300	7,219,285	T	G	G(T,G)	Similar to sugar carrier protein C
		7,219,715	A	G	M(A) / V(G)	
36	Os09g0297400	7,222,278	C	T	-	Similar to Phosphate/phosphoenolpyruvate translocator
		7,222,500	C	G	A(C,G)	
		7,224,772	C	T	-	
		7,225,524	C	T	-	
37	Os09g0297800	7,232,112	G	A	A(C) / D(A) / G(G)	Similar to GPI-anchored protein.
		7,232,427	A	T	-	
38	Os09g0297900	7,233,528	C	T	P(C) / L(T)	Conserved hypothetical protein
39	Os09g0298100	7,240,638	C	T	I(C,T)	Similar to predicted protein
40	Os09g0298266	7,246,438	A	T	-	Hypothetical gene
41	Os09g0298332	7,262,928	A	G	-	Conserved hypothetical protein
		7,263,028	C	A	P(C) / T(A)	
		7,263,030	G	A	P(G,A)	
		7,263,083	G	A	R(G) / H(A)	
42	Os09g0298400	7,271,375	G	A	-	WD40/YVTN repeat-like domain containing protein
		7,271,467	C	G	D(C) / E(G)	
		7,271,882	A	G	-	
42	Os09g0298400	7,271,978	C	A	-	WD40/YVTN repeat-like domain containing protein
		7,272,245	T	C	M(T) / T(C)	
43	Os09g0298700	7,293,500	T	C	R(G) / P(C) / L(T)	Nucleotide-binding 2C alpha-beta plait domain containing protein
		7,296,497	T	A	E(A) / D(T)	

		7,296,539	G	A	L(A,G)	
		7,296,619	A	G	R(G) / K(A)	
		7,296,674	T	C	H(T,C)	
		7,296,789	T	C	G(A,C,T)	
		7,297,117	T	G	R(G) / M(T)	
		7,297,151	C	T	P(A,T,C)	
		7,297,667	C	T	-	
		7,298,025	A	G	Q(C,G) / K(A)	
		7,298,385	T	C	Q(C) / -(T)	
		7,298,620	C	T	Q(A) / L(T) / P(C)	
		7,299,100	G	A	S(C) / V(A) / C(G)	
44	Os09g0299200	7,329,697	A	C	-	MYB-type transcription factor
		7,331,515	G	A	G(G) / E(A)	
45	Os09g0299400	7,336,763	T	C	V(C,T)	Similar to TPK1
		7,336,832	G	A	A(G,A)	
		7,337,153	C	T	L(C,T)	
		7,337,226	C	G	G(G) / R(C)	
		7,337,258	T	G	R(G,T)	
		7,337,382	C	A	M(A) / L(C)	
		7,338,873	G	A	-	
		7,339,466	T	C	-	
		7,339,580	A	G	-	
		7,339,897	C	G	-	
		7,340,117	C	A	I(A) / L(C)	
45	Os09g0299400	7,340,198	C	T	-	Similar to TPK1
46	Os09g0299500	7,347,460	A	G	-	Similar to predicted protein
		7,347,709	C	T	-	
		7,347,940	T	C	-	
		7,348,135	C	A	G(G) / S(A) / R(C)	
47	Os09g0301800	7,519,094	G	A	-	Hypothetical conserved gene
		7,520,853	A	G	N(A) / S(G)	
		7,521,294	C	T	T(C,T)	
		7,521,988	A	T	L(A,T)	
		7,522,797	A	G	-	
48	Os09g0302233	7,537,286	G	T	-	Similar to Zinc knuckle domain-like
49	Os09g0304400	7,741,337	T	C	M(G) / I(C,T)	Conserved hypothetical protein
		7,742,719	A	G	E(A,G)	

Table S3. Genotype information of 96 KASP markers for background selection.

Name	Chromosome	Position(bp)	Samgwang	Milkyqueen	Name	Chromosome	Position(bp)	Samgwang	Milkyqueen
KJ01_001	1	728,809	B	A	KJ04_095	4	35,078,228	B	A
KJ01_015	1	4,209,102	A	B	KJ05_005	5	1,243,736	B	A
KJ01_031	1	9,880,602	B	A	KJ05_015	5	5,360,210	B	A
KJ01_045	1	13,519,853	B	A	KJ05_022	5	7,194,845	A	B
KJ01_063	1	19,095,472	A	B	KJ05_028	5	11,098,186	A	B
KJ01_069	1	21,109,231	B	A	KJ05_030	5	15,317,821	B	A
KJ01_082	1	27,055,210	B	A	KJ05_039	5	18,623,022	A	B
KJ01_088	1	30,307,657	A	B	KJ05_051	5	22,081,477	A	B
KJ01_100	1	34,102,017	A	B	KJ05_060	5	24,999,987	B	A
KJ01_113	1	38,104,765	B	A	KJ05_076	5	28,718,002	A	B
KJ01_130	1	43,140,530	B	A	KJ06_004	6	637,769	A	B
KJ02_001	2	3,548,115	A	B	KJ06_014	6	3,977,139	A	B
KJ02_005	2	4,656,692	A	B	KJ06_027	6	7,708,089	B	A
KJ02_011	2	5,819,810	A	B	KJ06_040	6	10,780,360	A	B
KJ02_020	2	9,978,736	B	A	KJ06_047	6	13,351,589	A	B

KJ02_030	2	20,079,102	B	A	KJ06_064	6	17,875,066	A	B
KJ02_065	2	29,958,026	B	A	KJ06_071	6	21,799,623	B	A
KJ02_071	2	32,118,114	B	A	KJ06_079	6	24,997,051	A	B
KJ02_073	2	33,142,844	A	B	KJ06_082	6	29,179,807	A	B
KJ02_081	2	35,187,201	A	B	KJ07_017	7	3,705,757	B	A
KJ03_004	3	962,390	B	A	KJ07_026	7	7,431,843	B	A
KJ03_010	3	2,745,334	A	B	KJ07_033	7	11,030,430	A	B
KJ03_016	3	4,788,297	B	A	KJ07_039	7	15,311,521	A	B
KJ03_022	3	6,830,072	B	A	KJ07_061	7	20,728,990	B	A
KJ03_036	3	17,273,847	A	B	KJ07_075	7	24,968,707	B	A
KJ03_050	3	20,832,905	A	B	KJ07_082	7	28,377,055	B	A
KJ03_060	3	24,294,753	B	A	KJ08_011	8	2,940,939	A	A
KJ03_067	3	28,167,520	A	B	KJ08_026	8	6,404,031	A	B
KJ04_004	4	864,053	B	A	KJ08_038	8	9,004,923	A	B
KJ04_027	4	5,789,508	A	B	KJ08_044	8	10,273,969	B	A
KJ04_040	4	9,411,427	A	B	KJ08_060	8	14,184,710	A	B
KJ04_048	4	12,116,458	B	A	KJ08_074	8	17,642,104	B	A
KJ04_053	4	17,250,626	B	A	KJ08_087	8	20,637,053	A	B
KJ04_062	4	20,495,719	A	B	KJ08_091	8	26,979,434	A	B
KJ04_077	4	24,780,440	A	B	KJ09_011	9	3,627,789	B	A
KJ04_087	4	31,581,963	B	A	KJ09_025	9	7,555,220	A	B
KJ09_039	9	11,439,570	B	A	KJ11_043	11	12,295,393	A	B
KJ09_054	9	14,978,933	B	A	KJ11_057	11	15,591,509	A	B
KJ09_069	9	18,818,854	A	B	KJ11_072	11	19,593,598	B	A
KJ09_076	9	20,688,681	B	A	KJ11_089	11	23,101,149	A	B
KJ10_001	10	493,487	A	B	KJ11_100	11	27,626,227	B	A
KJ10_014	10	3,833,548	A	B	KJ12_007	12	3,791,039	B	A
KJ10_017	10	14,166,643	B	A	KJ12_016	12	5,782,882	A	B
KJ10_027	10	17,508,129	A	B	KJ12_026	12	10,614,915	B	A
KJ10_039	10	20,358,666	A	B	KJ12_036	12	14,173,587	A	B
KJ10_046	10	21,931,430	B	A	KJ12_045	12	16,493,747	B	A
KJ11_007	11	2,831,357	A	B	KJ12_055	12	21,484,803	A	B
KJ11_024	11	7,461,078	A	B	KJ12_062	12	25,427,169	B	A

Table S4. Statistical analysis for the segregation ratio of the selected lines in BC_2F_1 was detected as the segregation ratio of 1 : 2 : 1 by a χ2-test (P= 0.05) as expected in BC_2F_2 between Samgwang and Milkyqueen.

**Population**	**Generation**	**Number of plants**				**χ^2 value (1:2:1)**
		**Total**	**Homo (G/G)**	**Hetero (G/A)**	**Homo (A/A)**	
Samgwang x Milkyqueen	BC_2F_2	70	15	39	16	0.943^{ns}

d.f = 2: $\chi 2$ (0.05,2)=5.99

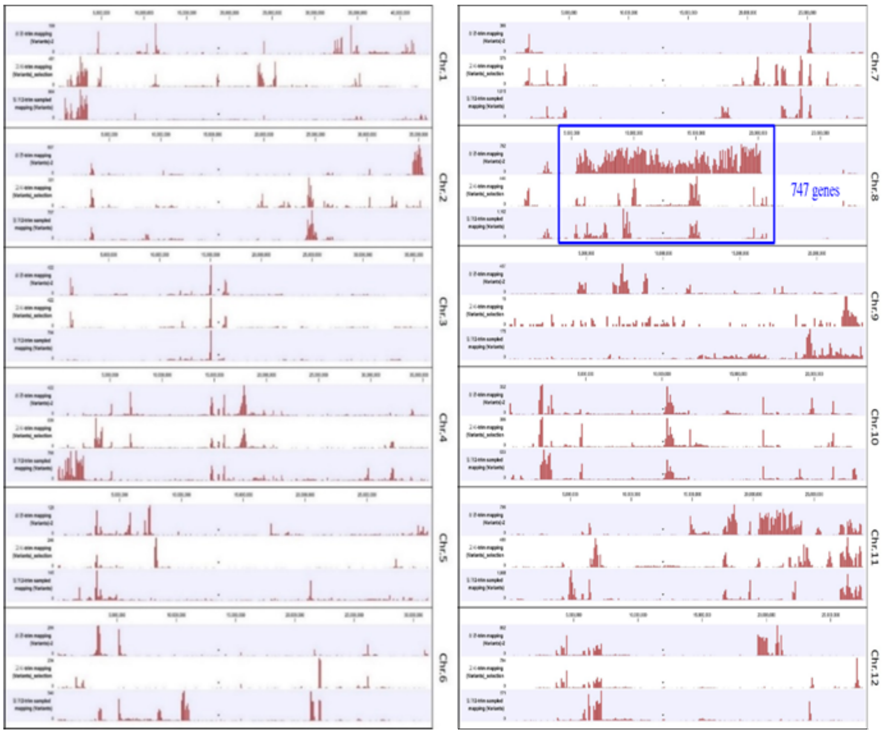

Figure S1. Distribution of SNPs between Samgwang and Milkyqueen, Kosihikari in the 12 rice chromosomes. The X-axis represents the physical distance along each chromosome in base-pair (bp) unit. The Y-axis indicates the common logarithm of the number of SNPs.

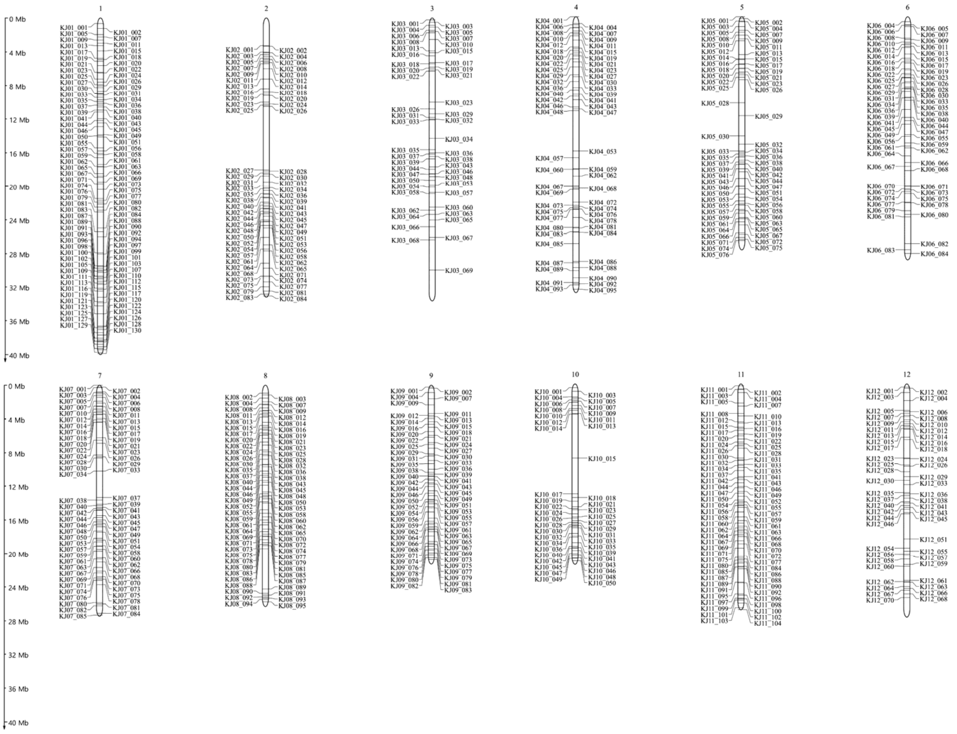

Figure S2. 773 KASP markers were used for analysis of marker effectiveness between parents, Samgwang and Milkyqueen.

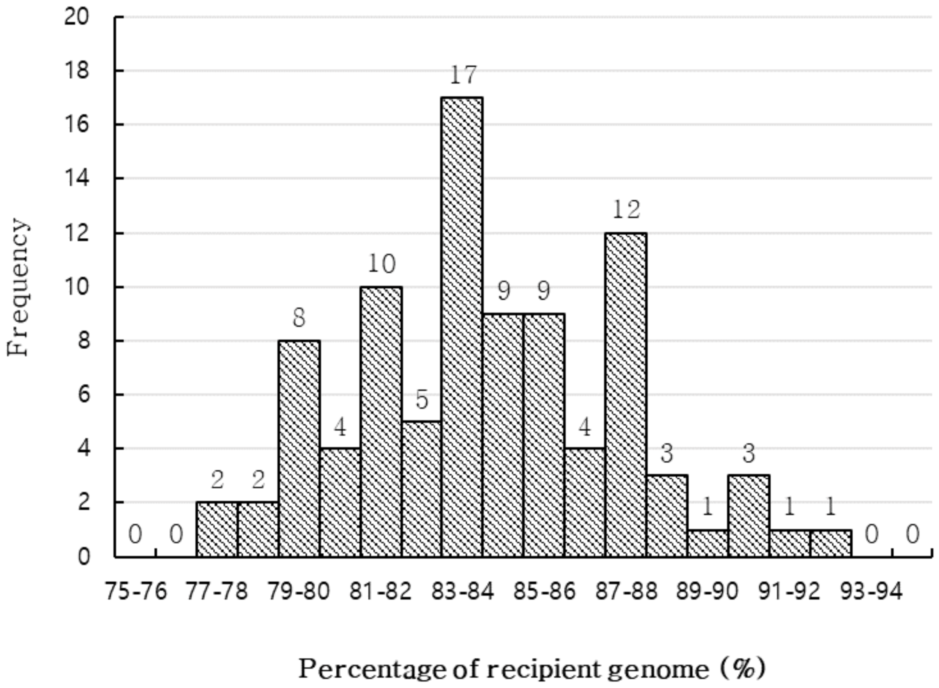

Figure S3. Frequency distribution of the genome recovery rate (%) of the recurrent parent in BC1F1 population.

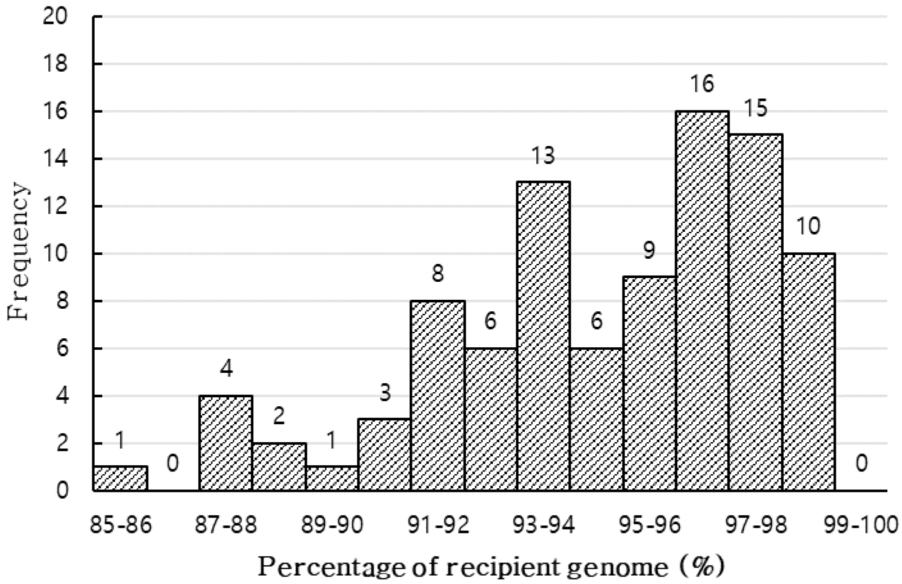

Figure S4. Frequency distribution of the genome recovery rate (%) of the recurrent parent in BC2F1 population.
